# Treatment Response and Tolerability of Pegylated Interferon-α Plus Ribavirin Combination Therapy in elderly Patients (≥ 65 years) With Chronic Hepatitis C in Korea

**DOI:** 10.5812/hepatmon.6170

**Published:** 2012-07-30

**Authors:** Hyeong Il Kim, In Hee Kim, Byung Jun Jeon, Seok Lee, Seong Hun Kim, Sang Wook Kim, Seung Ok Lee, Soo Teik Lee, Dae Ghon Kim

**Affiliations:** 1 Department of Internal Medicine, Chonbuk National University Medical School and Hospital, Jeonju, Jeonbuk, South Korea

**Keywords:** Chronic Hepatitis C, Ribavirin, Korea

## Abstract

**Background:**

The prevalence of hepatitis C virus (HCV) infections in elderly patients has been increasing in a number of countries. A few reports concerning pegylated interferon-α (PEG-IFN-α)-based combination treatment in elderly chronic hepatitis C (CHC) patients have been published, with slightly different treatment outcomes.

**Objectives:**

We investigated the treatment response and safety of PEG-IFN-α plus ribavirin combination therapy in elderly patients with CHC.

**Patients and Methods:**

Among a total of 181 treatment-naïve CHC patients (60 patients with genotype 1, 121 patients with genotype 2 or 3), 38 were aged ≥ 65 years (defined as the elderly group) and 143 were aged < 65 years (defined as the non-elderly group).

**Results:**

The overall sustained virologic response (SVR) was lower in the elderly group than in the non-elderly group, but it was not significantly different (65.8 % vs. 76.2 %, P = 0.15). In a subgroup analysis, among patients with genotype 1, the elderly group had a significantly lower SVR rate than the non-elderly group (30.8 % vs. 66.0 %, P = 0.03). However, the SVR rate in patients with HCV genotype 2 or 3 was comparable between the two groups (84.0 % vs. 81.3 %, P = 0.85). HCV genotype was significantly associated with SVR in the elderly patients (genotype 1 vs. 2 or 3, odds ratio: 0.18, 95% confidence interval: 0.000-0.869, P = 0.03). The incidence of premature discontinuation of treatment (21.1 % vs. 9.1 %, P = 0.05) and dose modification (52.6 % vs. 31.5 %; P = 0.02) due mainly to adverse events or laboratory abnormalities, were higher in the elderly group than in the non-elderly group.

**Conclusions:**

PEG-IFN-α plus ribavirin combination therapy might be considered for elderly CHC patients, especially for genotype 2 or 3, with vigilant monitoring of adverse events.

## 1. Background

Hepatitis C virus (HCV) infection is one of the main etiologies of chronic liver disease, with a global prevalence of 3 % [1]. It is estimated that approximately 85 % of patients with a HCV infection go on to develop chronic disease and up to 20 % will eventually develop cirrhosis, which may lead to liver failure, hepatocellular carcinoma (HCC), and death [2][3]. In Korea, the prevalence of HCV infection in the general population is approximately 1 % and this number increases with age peaking at 60 years or older [4]. The prevalence in older patients has been increasing in Korea, and this will become an impending problem in other countries where viral spread has occurred more recently as well. Thus, the care of aging patients with chronic hepatitis C (CHC) is one of the most important issues confronted by physicians. The current standard of care for CHC is a combination of pegylated-IFN-α (PEG-IFN-α) and ribavirin [5][6][7]. In pivotal clinical trials for the registration of PEG-IFN-α and ribavirin therapy, sustained virologic response (SVR), defined as an undetectable HCV RNA level 24 weeks after treatment completion, was achieved in 46 % of patients infected with HCV genotype 1, and in 82 % of cases with HCV genotypes 2 and 3 [8][9]. In those studies, older age was associated with a poorer response to treatment. However, it is not known whether the rate of response in persons older than 65 years is the same as or worse than that for persons 40–65 years of age, because these studies excluded patients > 65 years old. A few reports concerning PEG-IFN-α-based combination treatment in elderly CHC patients have been published, and these found slightly different treatment outcomes [10][11][12][13].

## 2. Objectives

The aim of this study was to investigate the treatment response and tolerability of PEG-IFN-α plus ribavirin combination therapy in elderly patients with CHC. We also assessed the factors associated with SVR in elderly patients.

## 3. Patients and Methods

### 3.1. Study Population and Study Design

We reviewed the medical records of CHC patients who had been treated with PEG-IFN-α plus ribavirin between May 2004 and December 2010, at the Chonbuk National University Hospital in Korea. Treatment-naïve adults of both sexes aged 18 years or more that met the following conditions were considered eligible; seropositivity for the HCV antibody (anti-HCV), quantifiable serum HCV RNA identified by real-time polymerase chain reaction (PCR) (COBAS AmpliPrep/COBAS TaqMan HCV test; Roche Molecular Systems, USA), and compensated liver disease. Exclusion criteria included; decompensated liver disease, uncontrolled depressive illness, drug or alcohol abuse within six months of study commencement, prior organ transplantation, positivity for hepatitis B virus or human immunodeficiency virus, pregnant status or an unwillingness/inability to comply with adequate contraception, untreated hyperthyroidism, autoimmune hepatitis, a hemoglobin level ≤ 10 g/dL, an absolute neutrophil count (ANC) ≤ 750/mm^3^, and a platelet count ≤ 50 000/mm^3^. Patients with known hypersensitivity to drugs used to treat HCV were also excluded. We recruited 191 patients who fulfilled the above criteria. Of these 191 patients, 10 patients who were undergoing current combination treatment or did not show any obvious effects of the antiviral treatment were also excluded. The data from the remaining 181 patients were finally analyzed. Study subjects received once weekly subcutaneous injections of either PEG-IFN-α-2a (Pegasys, Hoffmann-La Roche Ltd., Basel, Switzerland) 180 μg (79 patients) or PEG-IFN-α-2b (PegIntron, Shering Plough Corp., Kenilworth, NJ, USA) 1.5 μg/kg (for 24 weeks for HCV genotype 2, 3 or for 48 weeks for HCV genotype 1). In addition, all 181 participants received daily oral ribavirin; 800 mg/day for genotype 2 and 3 and 1 000 mg/day (body weight < 75 kg) or 1 200 mg/day (body weight ≥ 75 kg) for genotype 1. Patients aged ≥ 65 years were defined as the elderly group, and patients aged < 65 years were defined as the non-elderly group. Treatment response and adverse events were retrospectively compared between the two groups by an independent investigator unaware of study or treatment details. Informed consent was obtained from all study patients. This study was conducted in compliance with the World Medical Association Declaration of Helsinki and was approved by the Institutional Review Board at the Chonbuk National University Hospital.

### 3.2. Laboratory Tests

Baseline clinical and demographic characteristics, adverse events during treatment, and serum assay values (especially alanine aminotransferase (ALT), serum HCV RNA level, and HCV genotype) were stored in a database. HCV RNA was amplified by RNA PCR and hybridization methods (COBAS Amplicor HCV test, version 2.0; Roche Molecular Systems, Branchburg, NJ, USA; lower limit of detection 50 IU/mL), and the serum concentration of HCV-RNA was measured by real-time PCR (COBAS AmpliPrep/COBAS TaqMan HCV test; Roche Molecular Systems, USA). HCV genotyping was performed by reverse hybridization (INNO-LiPAHCV, Innogenetics, and Ghent, Belgium). A liver biopsy was not mandatory. In this study, liver cirrhosis was diagnosed mainly by ultrasonography (i.e., a coarse liver architecture, a nodular liver surface, and blunt liver edges) and evidence of hypersplenism (i.e., splenomegaly on ultrasonography and a platelet count of < 100 000/ mm^3^).

### 3.3. Treatment Response and Safety Assessments

Patients that received at least one dose of study medication were entered in the analysis, which was conducted on an intention-to-treat basis. The following variables were used to assess treatment response. Early virologic response (EVR) was defined as a ≥ 2 log_10_ reduction or complete absence of serum HCV RNA after 12 weeks of treatment versus baseline. End of treatment response (ETR) was defined as a single undetectable serum HCV RNA measurement by qualitative PCR at treatment completion. SVR was defined as a single undetectable serum HCV RNA measurement by qualitative PCR after 24 weeks of follow-up. Biochemical response (BR) was defined as serum ALT normalization after 24 weeks of follow-up. Relapse was defined as the reappearance of HCV RNA in the serum after treatment had been discontinued and ETR was documented. Drug discontinuation, dose modification, adverse events, and laboratory abnormalities were evaluated. Treatment was discontinued if adverse events were intolerable or if laboratory findings such as severe neutropenia (< 500/mm^3^) or anemia (hemoglobin < 6 g/dL) were abnormal in two consecutive samples. Dose modifications of PEG-IFN-α or ribavirin were implemented if neutropenia (< 750/mm^3^), anemia (hemoglobin < 10 g/dL), or thrombocytopenia (< 50 000/mm^3^) occurred; scheduled doses were re-administered after recovery. In addition, we assessed all of the visit records for the presence of adverse events such as flu-like symptoms (fever, headache, and myalgia), fatigue, anorexia/nausea, itching, rash, insomnia, alopecia, depression, or diarrhea.

### 3.4. Statistical Analysis

Results are reported as mean ± standard deviation (SD). Continuous variables were compared using the 2-tailed Student’s t-test and categorical data using the χ^2^ test or Fisher’s exact test. Factors associated with SVR were analyzed by univariate and multivariate analyses using a logistic regression model. P values < 0.05 were considered to be statistically significant. Data were transferred into a Microsoft EXCEL database (Microsoft Excel 2003) and analyzed using SPSS for Windows (version 15.0; SPSS Inc., Chicago, IL).

## 4. Results

### 4.1. Patient Baseline Characteristics

Among a total of 181 treatment-naïve CHC patients (60 patients with genotype 1, 121 patients with genotype 2 or 3), 38 were aged ≥ 65 years (elderly group) and 143 were aged < 65 years (non-elderly group). Among the 181 patients, 21 (8 elderly group; 13 non-elderly group) discontinued PEG-IFN-α plus ribavirin combination therapy due to adverse events or other reasons. The two groups were comparable with respect to the following demographic and baseline laboratory features; sex, presence of cirrhosis, baseline serum aspartate aminotransferase (AST) or ALT levels, AST: platelet ratio index (APRI) score, baseline serum HCV RNA level, and proportion of HCV genotypes (Table 1). Nine (23.7%) patients in the elderly group and 24 (16.8%) patients in the non-elderly group had compensated cirrhosis. Baseline serum HCV RNA levels were 5.6 ± 0.8 log_10_ IU/mL in the elderly group and 5.6 ± 0.8 log_10_ IU/mL in the non-elderly group. Of the 38 elderly patients, 13 (34.2 %) had HCV genotype 1 and 25 (65.8 %) had HCV 2 or 3 genotypes. Similarly, of the 143 non-elderly patients, 47 (32.9 %) had HCV genotype 1 and 96 (67.1 %) had HCV 2 or 3 genotypes. However, the elderly group had higher total bilirubin and lower albumin levels compared to the non-elderly group (0.8 ± 0.4 vs. 0.7 ± 0.3 mg/dL and 4.1 ± 0.4 vs. 4.2 ± 0.4 g/dL; P = 0.04 and 0.03, respectively).

**Table 1:** Baseline and Demographic Characteristics of Study Patients

	**Elderly (n = 38)**	**Non-Elderly (n = 143)**	**P value**
Age, y, mean ± SD	68.8 ± 4.4	49.5 ± 11.2	0.00
Female, No. (%)	14 (36.8)	67 (46.9)	0.36
Body mass index, mean ± SD	22.7 ± 2.9	23.7 ± 3.4	0.11
Cirrhosis, NO. (%)	9 (23.7)	24 (16.8)	0.35
Platelet count (× 103/mm^3^), mean ± SD	153.8 ± 53.9	167.1 ± 57.7	0.19
AST a, IU/L, mean ± SD	66.1 ± 37.5	73.0 ± 53.4	0.36
ALT^a^, IU/L, mean ± SD	71.9 ± 58.1	80.1 ± 68.9	0.46
Total bilirubin, mg/dL, mean ± SD	0.8 ± 0.4	0.7 ± 0.3	0.04
Albumin, g/dL, mean ± SD	4.1 ± 0.4	4.2 ± 0.4	0.03
APRI a score, mean ± SD	1.2 ± 0.8	1.3 ± 1.2	0.51
Serum HCV a RNA level, Log_10_ IU/mL, mean ± SD	5.6 ± 0.8	5.6 ± 0.9	0.91
**HCV genotype, No. (%)**			1.00
Type 1	13 (34.2)	47 (32.9)	
Type 2 or 3	25 (65.8)	96 (67.1)	

^a^ Abbreviations: ALT, Alanine Aminotransferase; APRI, platelet ratio index; AST, Aspartate Aminotransferase; HCV, hepatitis C virus

### 4.2. Treatment Responses

**Figure 1:**
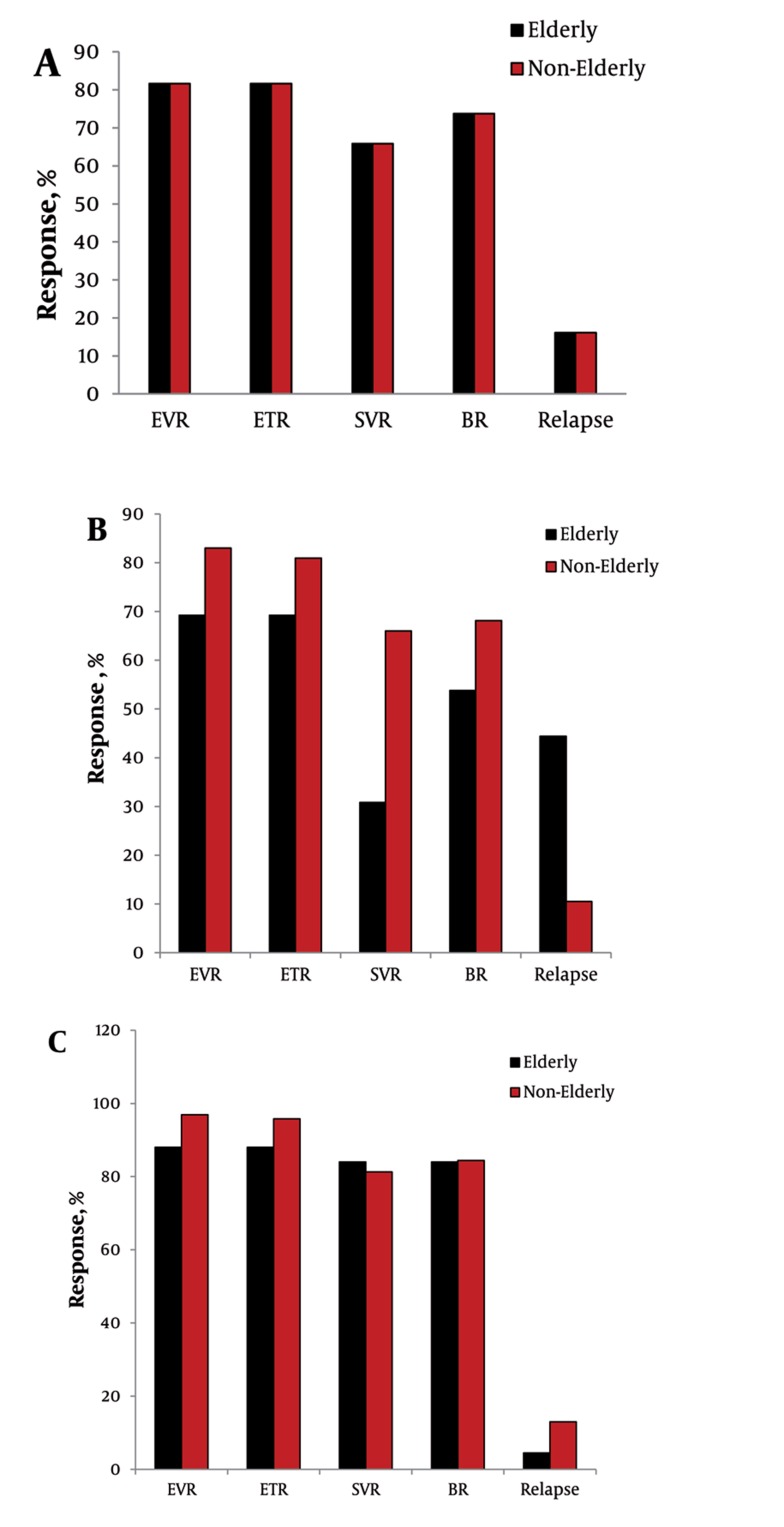
Treatment Response to PEG-IFN-α plus Ribavirin Therapy in Elderly versus Non-Elderly CHC Patients. (A) Overall (B) Patients with HCV genotype 1 (C) Patient with HCV genotype 2 or 3 EVR, early virologic response; ETR, end of treatment response; SVR, sustained virologic response; BR, biochemical response.

The overall treatment response and relapse rates are shown in Figure 1. The EVR, ETR, SVR, BR, and relapse rates of the elderly patients were comparable to those of the non-elderly group (81.6% vs. 92.3%, 81.6% vs. 90.9%, 65.8% vs. 76.2%, 73.7% vs. 79.0%, 16.1% vs. 12.3%; P = 0.07, 0.14, 0.15, 0.50, 0.56, respectively) (Figure 1A). In the subgroup analysis of patients with HCV genotype 1, the elderly group had a significantly lower SVR and higher relapse rate than the non-elderly group (30.8% vs. 66.0%, 44.4% vs. 10.5%; P = 0.03 and 0.03, respectively) (Figure 1B). However, SVR and relapse rate in patients with HCV genotype 2 or 3 were not significantly different between the elderly and non-elderly groups (84.0% vs. 81.3%, 4.5% vs. 13.0%; P = 0.90, 0.45, respectively) (Figure 1C).

### 4.3. Factors Associated with Sustained Virologic Response in Elderly Patients

To determine which baseline factors were predictive of SVR in the elderly patients, univariate and multivariate analyses were performed. HCV genotype (type 1 vs. type 2 or 3) was found to be significantly associated with increased SVR (P = 0.004) (Table 2). Other factors that were considered, but were not found to be significantly associated with SVR included; sex, body mass index, presence of cirrhosis, platelet count, baseline serum aminotransferase level, GGT, total bilirubin, albumin, total cholesterol, APRI score, and baseline serum HCV RNA level (≥ 800 000 IU/mL vs. < 800 000 IU/mL). Furthermore, multivariate analysis showed that only the HCV genotype was significantly associated with SVR (genotype 1 vs. 2 or 3, odds ratio: 0.18, 95 % confidence interval: 0.000-0.869, P = 0.03) (Table 3).

**Table 2:** Factors Associated With Sustained Virologic Response (SVR) in Elderly Patients by Univariate Analysis

	**Patients With SVR a (n = 25)**	**Patients Without SVRa (n = 13)**	**P value**
Female, No. (%)	8 (32.0)	5 (38.5)	0.57
Body mass index, mean ± SD	22.5 ± 2.8	23.2 ± 3.0	0.52
Cirrhosis, No. (%)	6 (24.0)	3 (23.1)	0.95
Platelet count (× 103/mm^3^), mean ± SD	161.0 ± 50.9	127.7 ± 48.1	0.07
AST a, IU/L, mean ± SD	63.2 ± 36.4	64.9 ± 34.5	0.89
ALT a, IU/L, mean ± SD	72.9 ± 56.3	58.1 ± 49.6	0.44
Albumin, g/dl, mean ± SD	4.1 ± 0.3	4.0 ± 0.5	0.28
APRI a score, mean ± SD	1.1 ± 0.8	1.3 ± 0.7	0.40
Baseline serum HCV RNA, Log_10_ IU/mL, mean ± SD	5.6 ± 0.9	5.7 ± 0.7	0.72
**Baseline serum HCV RNA, No. (%)**			0.56
≥ 800 000 IU/mL	12 (48)	7 (58.3)	
< 800 000 IU/mL	13 (52)	5 (46.2)	
**HCVa genotype, No. (%)**			0.004
Type 1	4 (16.0)	9 (69.2)	
Type 2 or 3	21 (84.0)	4 (30.8)	

^a^ Abbreviations: ALT, alanine amino transferase; APRI, platelet ratio index; AST, aspartate aminotransferase; HCV, hepatitis C virus; SVR, sustained virologic response

**Table 3:** Factors Associated With Sustained Virologic Response (SVR) in Elderly Patients by Multivariate Analysis.

	**Odds Ratio**	**95% CI**	**P value**
APRI a score	2.41	0.340 - 17.119	0.38
Baseline serum HCV RNA, Log10 IU/mL	0.25	0.009 - 6.759	0.41
Baseline serum HCV RNA (≥ 800 000 IU/mL vs. < 800 000 IU/mL)	2.31	0.016 - 335.46	0.74
HCV agenotype (type 1 vs. type 2 or 3)	0.18	0.000 - 0.869	0.04

^a^ Abbreviations: APRI, platelet ratio index; HCV, hepatitis C virus

### 4.4. Tolerability and Adverse Events

**Figure 2:**
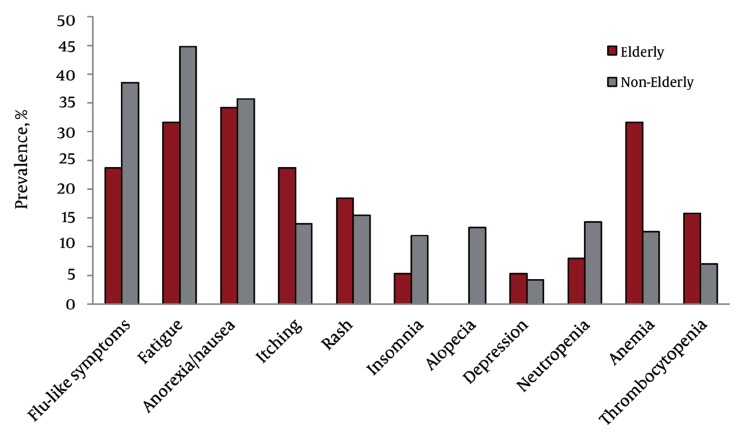
Adverse Events and Laboratory Abnormalities. Neutropenia, neutrophil counts ≤ 750 cells/mm^3^; anemia, hemoglobin level ≤ 10 g/dL; thrombocytopenia platelet counts ≤ 5×105 cells/mm^3^. All P values of adverse reactions other than alopecia and anemia were > 0.05

Of the 181 study subjects, 21 (11.6 %) showed premature discontinuation of treatment, which was more frequent in the elderly (21.1 %) than the non-elderly group (9.1 %) (P = 0.05) (Table 4). Furthermore, the rate of dose modification was significantly higher in the elderly than in the non-elderly group (52.6 % vs. 31.5 %; P = 0.016), especially the frequency of dose modification of ribavirin (34.2 % vs. 13.3 %; P = 0.003). The incidence of adverse events was not statistically different between the groups, but the incidence of alopecia was significantly higher in the non-elderly group (0 % vs. 13.3 % ; P = 0.02) (Figure 2). The incidence of neutropenia (≤ 750 cells/mm^3^) and thrombocytopenia (≤ 5 × 105 cells/mm^3^) were also not statistically different between the groups. However, the elderly group had a significantly higher incidence of anemia (< 10 g/dL) (31.6 % vs. 12.6 % ; P = 0.01) (Figure 2).

**Table 4:** Treatment Discontinuation and Dose Modification

	**Elderly Group, No. (%) (n = 38)**	**Non-Elderly Group, No. (%) (n = 143)**	**P value**
Premature discontinuation	8 (21.1)	13 (9.1)	0.05
Adverse events	5 (13.2)	8 (5.6)	1.00
Laboratory abnormalities	2 (5.3)	3 (2.1)	1.00
Other causes	1 (2.6)	2 (1.4)	1.00
Dose modification	20 (52.6)	45 (31.5)	0.02
PEG-IFN-α	11 (28.9)	40 (28)	0.91
Ribavirin	13 (34.2)	19 (13.3)	0.003

## 5. Discussion

It is generally expected that elderly patients will experience adverse events more frequently and have poorer treatment adherence, leading to inferior treatment efficacy. In this study, the elderly patients (≥ 65 years) with HCV genotype 1 showed significantly lower SVR and higher relapse rate than the non-elderly patients. However, the SVR rates of the elderly patients with genotype 2 or 3 were comparable to those of younger patients. HCV genotype was significantly associated with SVR. In addition, the incidence of premature treatment discontinuation and dose modification were higher in the elderly group than in the non-elderly group. Previous studies from Japan have shown that the SVR rates of PEG-IFN-α and RBV therapy were significantly lower among elderly patients [10][11]. Honda et al. reported that the SVR rate was significantly lower in elderly patients (≥ 65 years) compared to younger patients (< 65 years), (37.4 % vs. 51.5 % , P = 0.01), viral load and genotype were significantly associated with SVR in elderly patients, and female patients were less likely to achieve SVR than male patients [10]. Kainuma et al. also reported that the SVR rate decreased significantly with age in all genotypes and was markedly reduced in genotype 1 [11]. Among patients with genotype 1, the SVR rate was significantly lower in patients 65 years or older than in those less than 65 years (22.7 % vs. 47.3 % , P < 0.001). In addition, among patients with genotype 2, the SVR rate was significantly lower in patients 65 years or older than in those less than 65 years (65.6 % vs. 82.9 % ) (P = 0.004). However, a recent study by Nishikawa H et al. reported that there was no significant difference in the sustained virological response rate between younger patients and elderly patients (≥ 65 years) according to their hepatitis C virus genotype (41.5 % vs. 40.7 % for genotype 1; P = 0.899, 89.7 % vs. 86.4 % for genotype 2; P = 0.703, respectively) [12]. They suggested that obtaining an early virological response may be essential to achieve a sustained virological response in elderly patients with a CHC infection. Furthermore, a Korean study by Sinn et al. showed that older patients had a SVR rate that was comparable to the SVR rates of younger patients (80 % , 73 % , and 75 % for the < 50 years, 50-59 years, and ≥ 60 years patient groups, respectively; P = 0.42) [13]. However, Sinn et al. defined older patients as 60 years or older, which was lower than patients in previous studies from Japan, and the percentage of genotype 1 patients was lower than in the study by Honda et al. (40 % vs. 81 % ) [10][12]. In addition, the APRI was found to be the only independent predictor of SVR, translating to 95 % of the elderly patients with an APRI < 0.80 [13]. In our study, the overall SVR was not significantly different between the elderly (≥ 65 years) and non-elderly patients (< 65 years) (65.8 % vs. 76.2 % , P = 0.15). However, elderly patients with genotype 1 showed significantly lower SVR than non-elderly patients (30.8 % vs. 66.0 % , P = 0.03). In contrast, the SVR rate in patients with HCV genotype 2 or 3 was not significantly different between the elderly and non-elderly groups (84.0 % vs. 81.3 % , P = 0.85). In this study, HCV genotype was the only independent factor associated with SVR in the elderly patients (P = 0.04), while viral load and APRI score were not significantly associated with SVR. The discrepancies in factors associated with SVR may be due to the relatively small sample size of the elderly group and the possibility of bias as better candidates were more likely to be selected among the older compared to the younger patients. Consistent with previous studies [10][11][13], we found that the rates of treatment discontinuation and dose modification due to adverse events or laboratory abnormalities were higher for older patients. Interestingly, our study showed that the elderly group had a significantly higher incidence of anemia (< 10 g/dL) (31.6 % vs. 12.6 % ; P = 0.005) and more frequent dose modification of ribavirin (34.2 % vs. 13.3 % ; P = 0.003). Because a substantial amount of the ribavirin dose is excreted by the kidney, the risk of toxic reactions to this drug may be greater for elderly patients in whom the serum creatinine level might remain normal as the glomerular filtration rate decreases [14]. Therefore, ribavirin should be administered cautiously to elderly patients, starting at the lower end of the dosage range, with renal function monitoring and dosage adjustments made as needed. A common adverse effect of ribavirin is reversible hemolytic anemia, and the risk increases with age [15][16]. Dose reduction is recommended when the hemoglobin level is < 10 g/dL, and therapy cessation is recommended when the hemoglobin concentration decreases to < 8.5 g/dL [7].

CHC is becoming a disease of the elderly and elderly patients with chronic HCV infection often being a severe and underestimated disease. An Italian study by Gramenzi A et al. reported that elderly patients (≥ 65 years) more likely to had advanced liver disease (liver cirrhosis or hepatocellular carcinoma) compared with younger patients (51 % vs. 26 % ; P < 0.0001), but only 20 % of the elderly patients had received antiviral treatment [17]. Although elderly patients are considered and referred for treatment less often, current treatment guidelines do not suggest an age limitation for antiviral therapy [5][6][7]. Treatment decisions for older patients should be individualized, based on; the severity of liver disease, potential for serious adverse effects, likelihood of treatment response, and presence of co-morbid conditions. Regarding treatment, only patients who have more severe liver involvement than portal fibrosis, with at least moderate inflammation and necrosis and a significant risk of liver cirrhosis during their estimated life expectancy should be considered candidates for therapy. Therapy is contra-indicated for patients with decreased life expectancy due to; severe hypertension, heart failure, or coronary artery disease and for patients with poorly controlled diabetes or obstructive lung disease [5][6][7]. We note that this study is intrinsically limited by its retrospective, single center design and by the small size of the elderly group. Furthermore, our investigation of adverse events was limited to medical records. Nevertheless, we believe that this study offers valuable information regarding the therapeutic efficacy and tolerability of PEG-IFN-α and ribavirin combination therapy for elderly patients with CHC in Korea. A further large scale, multicenter study is warranted to confirm our results.

In conclusion, we found that the SVR rate of PEG-IFN-α plus ribavirin combination therapy was lower in elderly patients (≥ 65 years) with genotype 1. However, the SVR rate among elderly patients with HCV genotype 2 or 3 was comparable to those of non-elderly patients. The HCV genotype was independently associated with SVR. Accordingly, we suggest that PEG-IFN-α plus ribavirin combination therapy might be considered for elderly patients, especially for patients with genotype 2 or 3, with cautious monitoring of adverse events.
